# Effects of bedtime periocular and posterior cervical cutaneous warming on sleep status in adult male subjects: a preliminary study

**DOI:** 10.1007/s41105-017-0129-3

**Published:** 2017-10-14

**Authors:** Michihito Igaki, Masahiro Suzuki, Ichiro Sakamoto, Tomohisa Ichiba, Kenichi Kuriyama, Makoto Uchiyama

**Affiliations:** 1 Personal Health Care Laboratory, Kao Corporation, 2-1-3, Bunka, Sumida-ku, Tokyo, 131-8501 Japan; 2 Department of Psychiatry, Nihon University School of Medicine, Oyaguchi Kamicho, Itabashi-ku, Tokyo, 173-8610 Japan; 3 Department of Psychiatry, Shiga University of Medical Science, Seta Tsukinowa-cho, Otsu, Shiga 520-2192 Japan

**Keywords:** Periocular cutaneous warming, Posterior cervical cutaneous warming, Heat and steam-generating sheet, Subjective sleep status, Objective sleep quality, Delta power density

## Abstract

Appropriate warming of the periocular or posterior cervical skin has been reported to induce autonomic or mental relaxation in humans. To clarify the effects of cutaneous warming on human sleep, eight male subjects with mild sleep difficulties were asked to try three experimental conditions at home, each lasting for 5 days, in a cross-over manner: warming of the periocular skin with a warming device for 10 min before habitual bedtime, warming of the posterior cervical skin with a warming device for 30 min before habitual bedtime, and no treatment as a control. The warming device had a heat- and steam-generating sheet that allowed warming of the skin to 40 °C through a chemical reaction with iron. Electroencephalograms (EEGs) were recorded during nocturnal sleep using an ambulatory EEG device and subjected to spectral analysis. All the participants reported their sleep status using a visual analog scale. We found that warming of the periocular or posterior cervical skin significantly improved subjective sleep status relative to the control. The EEG delta power density in the first 90 min of the sleep episode was significantly increased under both warming of the periocular or posterior cervical skin relative to the control. These results suggest that warming of appropriate skin regions may have favorable effects on subjective and objective sleep quality.

## Introduction

Reportedly approximately 20% of the general adult population in Japan has difficulty sleeping [1], being comparable to data reported elsewhere [2]. Studies have shown that sleep problems are associated with poor daytime function, and are a risk factor for physical or mental disorders, thus raising awareness of the importance of sleep as a public health issue [1–4]. Hypnotic drug therapy for insomnia has been widely employed, but its long-term use may be problematic in terms of untoward effects and dependence issues [5]. Sleep hygiene education and cognitive behavior therapy for insomnia (CBT-I) have been recommended as alternative behavioral and psychological therapeutic options [6, 7].

As a physiological intervention, bathing at an appropriate temperature and with optimal timing has been reportedly associated with a subjective improvement of sleep initiation and an objective increase in deep EEG sleep, possibly due to general relaxation and/or increased heat loss after such warming interventions [8–10]. Warming of the hand or foot skin before bedtime has been reported to improve sleep quality and decrease core body temperature in elderly subjects with or without insomnia [11, 12].

We have developed a disposable heat- and steam-generating sheet to warm the periocular skin safely and easily, and reported that it reduced sympathetic and increased parasympathetic nerve activity [13] and enhanced alpha-band electroencephalogram activity during the daytime [14], leading to autonomic or mental relaxation.

We postulated that this heat- and steam-generating sheet might have potential application for improvement of poor sleep through warming of the skin and autonomic relaxation. In the present study, we examined the effect of periocular and posterior cervical cutaneous warming with our disposable heat- and steam-generating sheet before bedtime. Using electroencephalography (EEG) and a visual analog scale (VAS), we evaluated objective and subjective sleep quality in subjects who had mild difficulty falling asleep.

## Materials and methods

### Participants

**Fig. 1:**
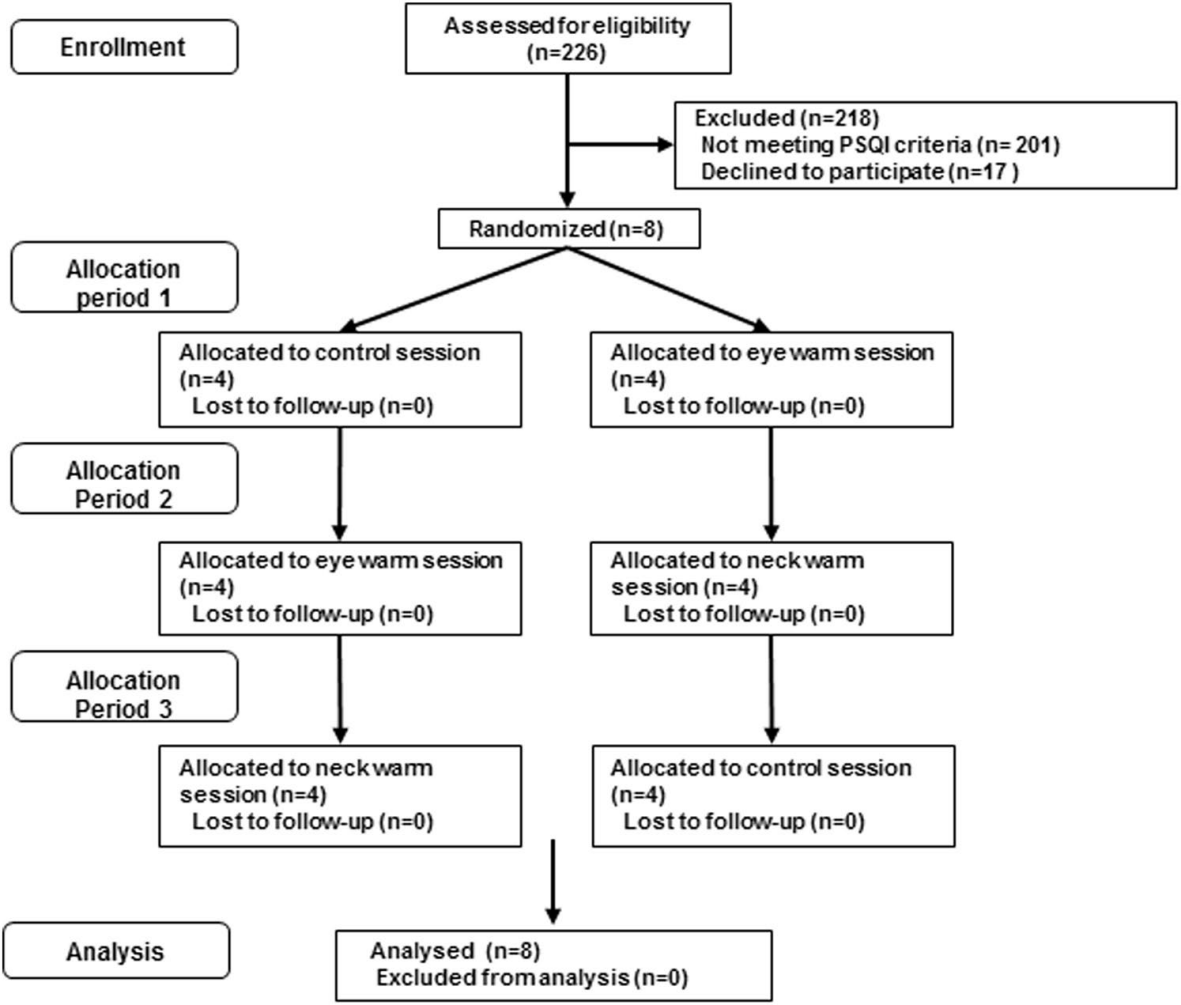
Subject flow chart

The experimental procedures were in accordance with the guidelines outlined in the Declaration of Helsinki and approved by the Ethics Committee of Nihon University (Approval number: 27-9). First, an intranet email, in which the objectives and procedures of the present study were concisely described, was sent to 678 male workers of Kao Co., aged between 20 and 59 years, in order to recruit the study participants. The participants included were (1) those who had experienced sleep difficulties scored as six or more in the Pittsburgh sleep questionnaire and who had found it difficult to fall asleep once or more per week in the previous month, but (2) who had not suffered daytime impairment of daily life or had taken hypnotic medication in the previous month. Two-hundred and twenty-six males responded via intranet and were interviewed by the investigators. We found that 25 males fulfilled the inclusion criteria. Finally, eight healthy males (mean age ± SD 38.5 ± 11.8 years) submitted their written informed consent after the possible risks and details of the study had been explained (Fig. 1).

### Experimental procedures

**Fig. 2:**
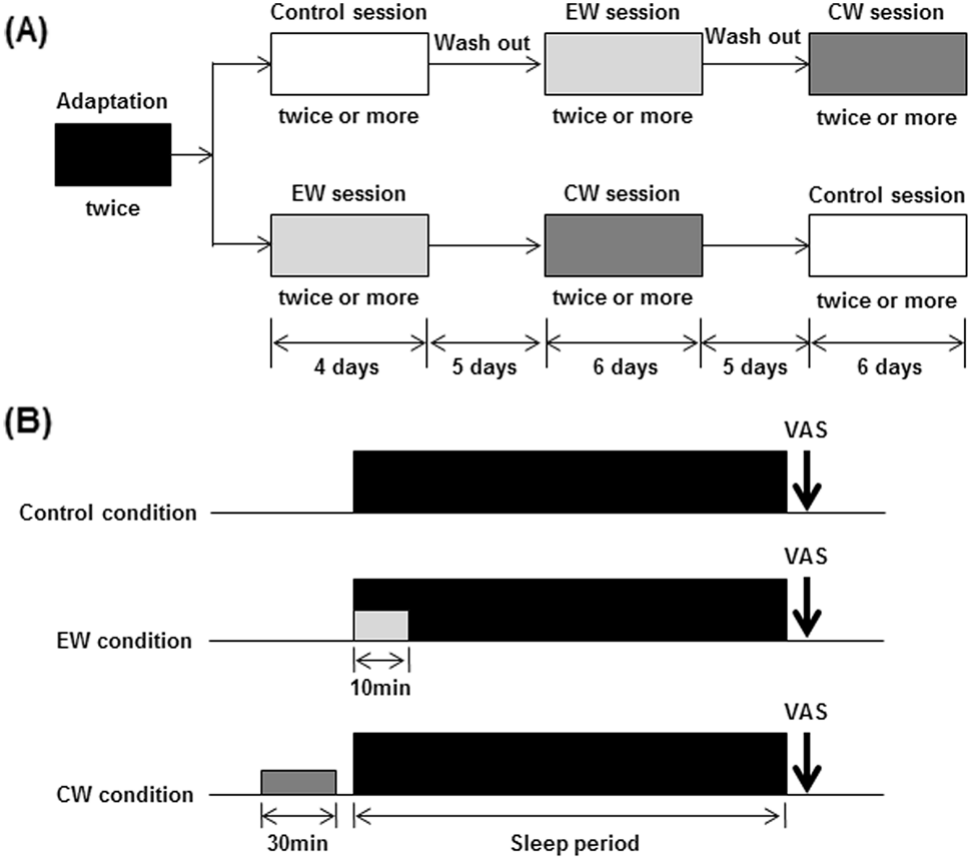
Experimental design. **a** Schedule proposal. **b** Typical sleep period for a day under EW and CW conditions. *EW* periocular cutaneous warming; *CW* posterior cervical cutaneous warming

Figure 2a illustrates the experimental schedules used in the present study. Each experiment was performed at the home of the participant. After a 7-day adaptation period, participants underwent three experimental sessions separated by a 5-day intersession interval: a control session without any interventions, a periocular cutaneous warming (EW) session using a disposable eyelid-warming device (EWD), and a posterior cervical cutaneous warming (CW) session using a disposable posterior cervical cutaneous-warming device (CWD). The subjects were required to maintain their habitual daily sleep-wake schedule throughout the experiment but were prohibited from drinking alcohol and taking intense physical exercise. Four subjects started with the control session and then took part in the EW and CW sessions, whereas the other subjects took part in the control session after the EW and CW sessions (Fig. 2a), thus, allowing the control session and two intervention sessions to be crossed over. The experimental sessions were scheduled to weekdays between January and March, 2013.

In the EW session, each participant was instructed to wear the EWD for 10 min from habitual bedtime. In the CW session, a 30-min warming period with the CWD was conducted in a sitting position just before bedtime (Fig. 2b).

All-night sleep electroencephalography from bedtime to waking time was conducted twice in the adaptation period using a newly developed small ambulatory EEG machine. Similarly, all-night sleep EEG was conducted twice or more in each of the experimental sessions (control, EW and CW sessions).

Both the warming devices (EWD and CWD) were made of non-woven fabric and had a disposable heat and steam-generating sheet, which provided moist heat through the chemical reaction of iron, water, and oxygen when the package was opened. The EWD warmed the periocular skin to 40 °C within approximately 10 min [15], and the CWD warmed the posterior cervical skin to approximately 40 °C within approximately 30 min. The EWD and CWD used in these conditions were reported to provide a significant relaxation in autonomic nervous system based on heart rate variability [16, 17].The EWD and CWD were the prototype made by Kao Corporation for the present study.

### Evaluation of subjective sleep status

Subjective sleep status for the control, EW, and CW conditions was assessed upon awakening using a 100-mm VAS for separate evaluation of restfulness, subjective refreshment, sleep initiation, recovery from fatigue and sleep quality, respectively.

### Electroencephalography during sleep

EEG was performed during sleep with an ambulatory sleep EEG apparatus (SLEEP SCOPE; Sleep Well Co., Japan) [18, 19], equipped with a single EEG channel. Using this apparatus, we obtained an EEG record from an electrode placed in the median frontal region referenced to a right mastoid electrode, and then subjected it to off-line analysis. In accordance with criteria originally described elsewhere [18, 19], the sleep EEG record was divided into 30-s epochs and classified into waking, rapid eye movement (REM) sleep, light non-REM sleep or deep non-REM sleep. Additionally, sleep latency, sleep efficiency, and wake after sleep onset were calculated. EEG data obtained by this apparatus have recently been reported in several studies [18, 19].

Spectral analyses of the EEG data were conducted using an automatic fast Fourier transform algorithm. The EEG power density for consecutive 90-min periods was calculated for the following frequency bands: delta (0.5–2.0 Hz), theta (4.0–8.0 Hz), alpha (8.0–12.0 Hz), sigma (12.0–16.0 Hz) and beta (16.0–35.0 Hz). Thus, the EEG power density for the first, second, and third 90-min periods was calculated. For individual records, the EEG power density of each session was averaged. The average number of times in each session was 2.9 ± 1.1 in control, 2.9 ± 1.2 in EW, and 2.6 ± 0.5 in CW session (average number ± SD).

### Statistical analyses

Values were expressed as means ± standard deviations of the mean (SD). Statistical analyses for comparisons between conditions were performed with the Wilcoxon signed-rank test. Probability values of less than 0.05 were accepted as statistically significant. All statistical analyses were performed using IBM SPSS Statistics 20 (IBM, Chicago, IL, USA).

## Results

### Sleep parameters and subjective sleep status

Table 1 shows time in bed (TIB), sleep period time (SPT), total sleep time (TST), sleep latency, sleep efficiency, and wake after sleep onset for the control, EW, and CW conditions. TIB, SPT, TST, sleep latency, sleep efficiency, and wake after sleep onset did not differ between the EW and control conditions, or between the CW and control conditions.

**Table 1:** Sleep parameters for experimental nights

	Control	EW	CW
Bedtime (hh:mm)	23:53 ± 0:15	23:48 ± 0:21	23:54 ± 0:14
Waketime (hh:mm)	5:59 ± 1:03	5:30 ± 0:45	5:39 ± 0:17
TIB (min)	385.8 ± 77.2	384.9 ± 29.5	361.9 ± 39.0
SPT (min)	375.5 ± 71.5	370.7 ± 23.1	354.7 ± 36.5
TST (min)	354.1 ± 64.3	352.6 ± 20.8	335.6 ± 35.2
Sleep latency (min)	9.6 ± 7.3	8.7 ± 6.4	6.3 ± 3.9
Sleep efficiency (%)	91.1 ± 2.9	92.2 ± 2.6	92.1 ± 2.6
Wake after sleep onset (min)	21.2 ± 8.6	15.9 ± 6.0	18.3 ± 5.7

Values are means ± SD

*EW* periocular cutaneous warming; *CW* posterior cervical cutaneous warming

Table 2 summarizes subjective sleep status upon awakening for the control, EW and CW conditions. Subjective refreshment, sleep initiation and sleep quality were significantly better for EW than for control conditions (Wilcoxon signed-rank test). Restfulness, subjective refreshment and sleep quality were significantly better for CW than for control conditions (Wilcoxon signed-rank test).

**Table 2:** Subjective sleep score in the morning

	Control	EW	*P*	CW	*P*
Feeling of restfulness (mm) (0: poor; 100: good)	47.1 ± 18.0	53.3 ± 15.1	n.s	62.3 ± 8.2	0.025
Feeling of being refreshed (mm) (0: poor; 100: good)	39.2 ± 16.2	53.8 ± 19.4	0.025	56.8 ± 7.7	0.017
Sleep initiation (mm) (0: poor; 100: good)	46.8 ± 20.2	72.0 ± 11.3	0.012	61.8 ± 21.3	n.s
Recovery from fatigue (mm) (0: low; 100: high)	47.6 ± 10.7	53.7 ± 14.8	n.s	53.1 ± 6.5	n.s
Sleep quality (mm) (0: low; 100: high)	38.8 ± 14.0	57.2 ± 16.8	0.025	59.3 ± 11.4	0.017

Values are means ± SD. Comparisons performed relative to control (Wilcoxon signed rank test)

*EW* periocular cutaneous warming; *CW* posterior cervical cutaneous warming

### EEG measurement

**Fig. 3:**
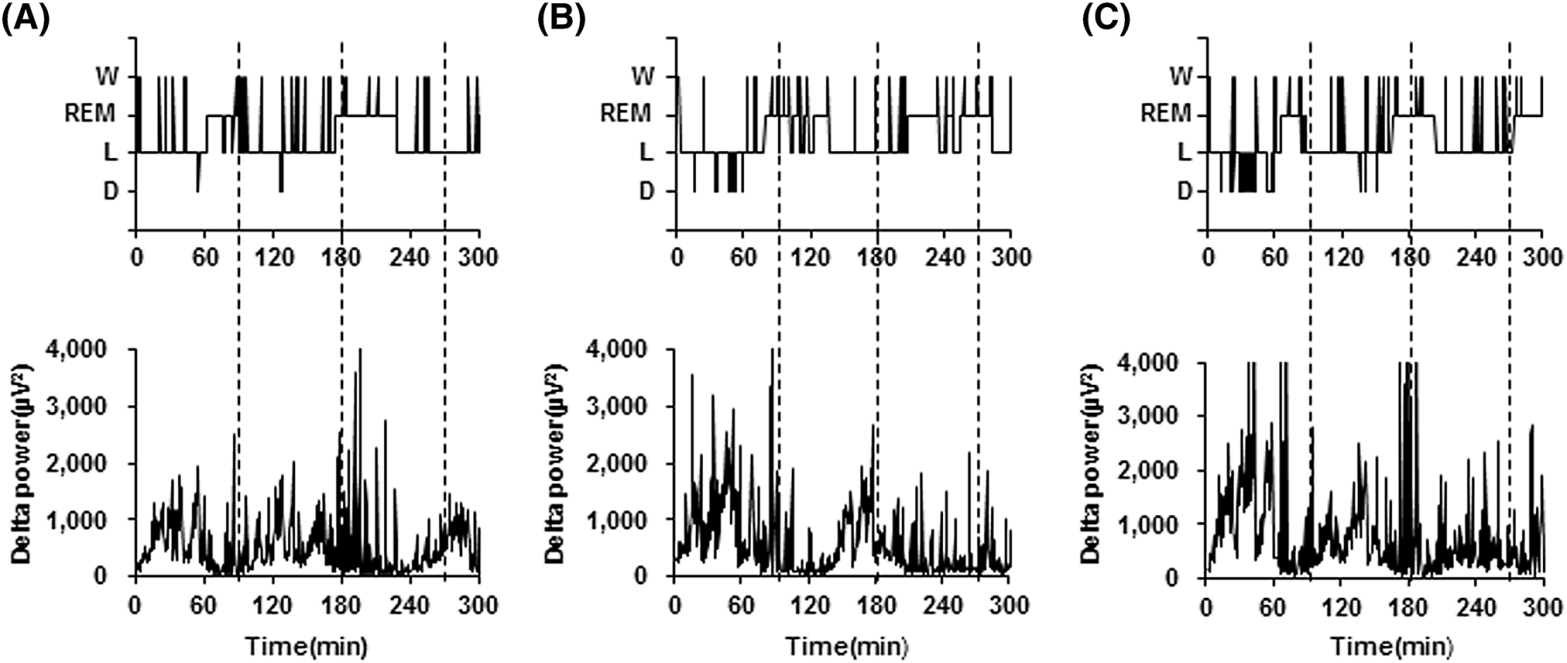
Profiles of the hypnogram (upper) and delta power (lower) under. **a** control, **b** EW and **c** CW conditions for a representative subject. *W* wake; *REM* rapid eye movement; *NREM* non-rapid eye movement; *L* NREM light sleep; *D* NREM deep sleep, *EW* periocular cutaneous warming; *CW* posterior cervical cutaneous warming

**Fig. 4:**
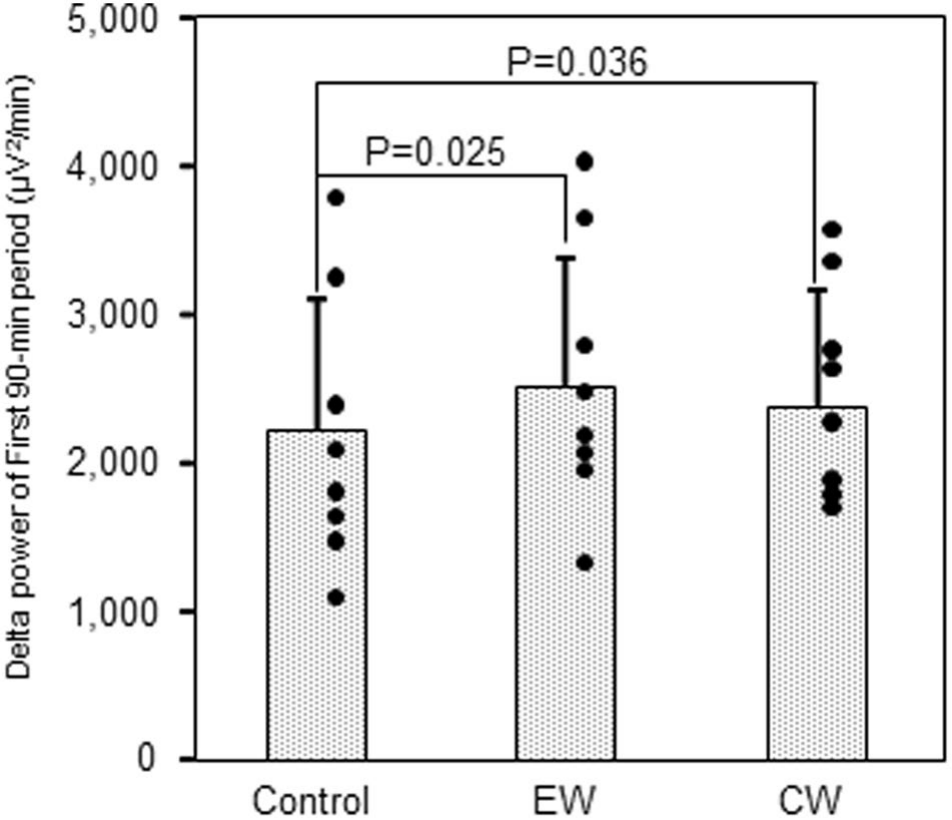
Delta power density in the first 90-min period. Scatterplot of delta power density in the first 90-min period in each condition. Individual data are shown. Values are means ± SD. Comparisons performed relative to control (Wilcoxon signed rank test). *EW* periocular cutaneous warming; *CW* posterior cervical cutaneous warming

Sleep EEG profiles for a representative subject are shown in Fig. 3. It was noted that the deep non-REM sleep stage and delta power in the first third of the sleep episodes were more marked under EW and CW conditions than under control conditions. Power densities obtained by EEG spectral analyses under control, EW and CW conditions were presented with respect to progression during the night (Table 3). In the first 90-min period, delta, theta and alpha power densities were significantly enhanced under EW conditions than under control conditions, while under CW conditions only the delta power density was enhanced relative to the control (Wilcoxon signed-rank test). In the second and third 90-min periods, none of the power densities for the 5 frequency bands (delta, theta, alpha, sigma, and beta) differed between EW and control conditions, or between CW and control conditions. The enhancements of delta power density in the first 90-min period are summarized in Fig. 4.

**Table 3:** Sleep EEG frequency power in each period between control and EW or CW conditions

EEG band	Control (μV^2^/min)	EW (μV^2^/min)	*P*	CW (μV^2^/min)	*P*
First 90-min period					
Delta power	2231 ± 885	2538 ± 857	0.025	2418 ± 772	0.036
Theta power	353 ± 100	406 ± 99	0.025	368 ± 94	n.s
Alpha power	183 ± 78	211 ± 87	0.025	175 ± 55	n.s
Sigma power	87 ± 43	73 ± 42	n.s	82 ± 29	n.s
Beta power	52 ± 31	57 ± 36	n.s	43 ± 23	n.s
Second 90-min period					
Delta power	1120 ± 363	1018 ± 375	n.s	1076 ± 272	n.s
Theta power	249 ± 79	290 ± 101	n.s	255 ± 76	n.s
Alpha power	142 ± 51	161 ± 71	n.s	143 ± 65	n.s
Sigma power	73 ± 37	69 ± 43	n.s	68 ± 28	n.s
Beta power	55 ± 27	49 ± 36	n.s	41 ± 23	n.s
Third 90-min period					
Delta power	1890 ± 739	2003 ± 775	n.s	1826 ± 635	n.s
Theta power	320 ± 129	374 ± 166	n.s	306 ± 88	n.s
Alpha power	163 ± 71	185 ± 93	n.s	153 ± 61	n.s
Sigma power	66 ± 34	62 ± 43	n.s	70 ± 32	n.s
Beta power	50 ± 26	55 ± 43	n.s	42 ± 26	n.s
Total					
Delta power	1716 ± 457	1858 ± 534	n.s	1778 ± 443	n.s
Theta power	328 ± 102	357 ± 120	0.025	323 ± 85	n.s
Alpha power	178 ± 70	194 ± 84	n.s	168 ± 67	n.s
Sigma power	79 ± 33	82 ± 49	n.s	77 ± 31	n.s
Beta power	57 ± 27	68 ± 42	n.s	45 ± 25	n.s

Values are means ± SD. Comparisons performed relative to control (Wilcoxon signed rank test)

*EW* periocular cutaneous warming; *CW* posterior cervical cutaneous warming

## Discussion

In the present study, warming of the bilateral periocular or posterior cervical skin before bedtime improved sleep subjectively and objectively in subjects having occasional difficulty falling asleep but without daytime consequences. Improvements in subjective refreshment and sleep quality were observed after either of these warming conditions. Subjective improvement in sleep initiation occurred after periocular cutaneous warming, while subjective improvement of restfulness occurred after posterior cervical cutaneous warming. Using the power density obtained from EEG spectral analyses as an objective sleep measure, we found that delta power in the first third of the sleep episodes was increased by both warming conditions. The present findings are the first to demonstrate that appropriate cutaneous warming of the periocular or posterior cervical region at around 40 °C improves sleep both subjectively and objectively.

There have been several reports on the effects of passive body heating on subjective and objective sleep. Studies using a variety of bathing conditions have suggested that bathing at an appropriate temperature (41 ± 0.5 °C) for 30 min before bedtime improves subjective sleep and increases slow wave sleep in the first sleep cycle [9, 10]. Both subjective and objective changes after passive body heating are likely to be related to sleep consolidation. Furthermore, warming of the hand or foot, being a more localized form of cutaneous temperature manipulation than bathing, has been reported to improve sleep in a similar manner [11, 12]. These effects of general or partial body heating in the previous studies seem to be comparable to the findings obtained in the present study.

Many studies have shown that appropriate warming of the skin on various parts of the human body at around 40 °C elicits a subjective feeling of comfort and/or relaxation [16, 20–22]. Some of these studies [13, 16, 17] conducted a simultaneous autonomic nervous system evaluation based on heart rate variability and found that periocular or cervical cutaneous warming reduced sympathetic and increased parasympathetic nerve activity, leading to potential reduction of somatic arousal. In this regard, the improved sleep observed in the present study after warming of the periocular or posterior cervical skin may be interpreted as a consequence of psychophysiological relaxation.

The effects of cutaneous warming on sleep in the present study may be also interpreted as a direct effect on body temperature. Delta power during sleep has been used as an objective marker of sleep consolidation, which is achieved by homeostatic elevation of sleep pressure [23]. Many studies have reported that various degrees of sleep deprivation increased delta power in the recovery night [24]. Anders et al. reported that the increase of delta power in the early hours of natural nocturnal sleep was associated with decrease of core body temperature [25]. Most experimental studies have indicated that the increase of delta power in the first few hours of nocturnal sleep is associated with the decline of core body temperature [26], while association between delta power and core body temperature in the middle or late hours of nocturnal sleep was not clearly demonstrated. The mechanism linking cutaneous temperature manipulation and sleep in first few hours after sleep onset may be promotion of heat dissipation and a reduction of core body temperature following changes in cutaneous temperature [27]. In the present study, we used a disposable heat- and steam-generating sheet that safely and easily warmed the skin of the periocular or posterior cervical region. Although we did not evaluate the core body or cutaneous temperature, it is possible to postulate that delta power was increased and subjective sleep status may have been improved through a similar mechanism, as a decrease of core body temperature due to heat loss in early hours of sleep has been reported in previous studies. Since the two areas which were used in EW and CW in the present study are anatomically close to the brain, the potential effects of direct warming of the brain through adjacent tissues could be considered as those demonstrated in animal studies [27–29]. However, we assumed that such effects were not likely in the present study because cutaneous temperature under the EWD and CWD was controlled around 40 °C even after the warming period of 10 and 30 min, respectively.

There were several limitations to the present study. The participants belonged to Kao Corporation, which held Personal Health Care Laboratory, the affiliation of three of the authors. Therefore, there could have been potential biases on the subjective measures of the study, while the main findings of the study were obtained from the spectral EEG analyses, which did not seem to be biased by the social characteristics of the participants. The experiments were carried out under home conditions, while participants maintained their habitual sleep-wake schedule and sleep environment. Therefore, potential confounding factors, such as room temperature, humidity, illumination and noise level, individual preference for environmental conditions (i.e., preference for hot or cold environment), were not completely controlled. Further investigation will be necessary to clarify the effects of periocular cutaneous warming and posterior cervical cutaneous warming under various environmental conditions. A multi-channel EEG device was usually used to collect EEG data for the entire night. For these data, sleep stages were determined visually according to the standard criteria. However, a single-channel portable EEG device was used, and EEG measurements were performed by the participants themselves. Therefore the EEG region might have been a confounding factor that changed the delta power density.

## Conclusion

This study has shown that the warming of the periocular or posterior cervical skin at around 40 °C before bedtime improved subjective refreshment and sleep quality in subjects having occasional difficulty falling asleep but without daytime consequences. The objective EEG delta power density in the first 90 min of the sleep episode was significantly increased under both warming conditions relative to the control. These results suggest that warming of appropriate cutaneous regions may have favorable effects on subjective and objective sleep quality.

## References

[1] Uchiyama M, Inoue Y, Uchimura N, Kawamori R, Kuribayashi M, Kario K (2011). Clinical significance and management of insomnia. Sleep Biol Rhythms.

[2] Ohayon MM (2002). Epidemiology of insomnia: what we know and what we still need to learn. Sleep Med Rev.

[3] Banks S, Dinges DF (2007). Behavioral and physiological consequences of sleep restriction. J Clin Sleep Med.

[4] Phillips B, Mannino DM (2007). Do insomnia complaints cause hypertension or cardiovascular disease?. J Clin Sleep Med.

[5] Buysse DJ, Insomnia (2013). JAMA.

[6] Morin CM, Kryger MH, Roth T, Dement WC (2010). Psychological and behavioral treatments for insomnia I: approaches and efficacy. Principles and practice of sleep medicine.

[7] Bertisch SM, Welles RE, Smith MT, McCarthy EP (2012). Use of relaxation techniques and complementary and alternative medicine by American adults with insomnia symptoms: results from a national survey. J Clin Sleep Med.

[8] Liao WC (2002). Effects of passive body heating on body temperature and sleep regulation in the elderly: a systematic review. Int J Nurs Stud.

[9] Bunnell DE, Agnew JA, Horvan SM, Jopson L, Wills M (1998). Passive body heating and sleep: Influence of proximity to sleep. Sleep.

[10] Silva A, Queiroz SS, Andersen ML, Mônico-Neto M, Campos MS, Roizenblatt S (2013). Passive body heating improves sleep patterns in female patients with fibromyalgia. Clinics.

[11] Kräuchi K, Cajochen C, Werth E, Wirz-Justice A (2000). Functional link between distal vasodilation and sleep-onset latency?. Am J Physiol Regul Integr Comp Physiol.

[12] Raymann RJEM, Swaab DF, Van Someren EJW (2007). Skin temperature and sleep-onset latency: changes with age and insomnia. Physiol Behav.

[13] Nagashima Y, Igaki M, Yada Y, Suzuki T, Oishi S (2006). Effect on autonomic nervous activity of the application of heat- and steam-generating sheets to the eyes. Auton Nerv Syst.

[14] Ochiai R (2001). Moist heat stimulation influence on electroencephalograms and the autonomic nervous system. Auton Nerv Syst.

[15] Takahashi Y, Igaki M, Sakamoto I, Suzuki A, Takahashi G, Dogru M (2010). Comparison of effects of periocular region dry and wet warming on visual acuity and near reflex. Nippon Ganka Gakkai Zasshi.

[16] Takamoto K, Hori E, Urakawa S, Katayama M, Nagashima Y, Yada Y (2013). Thermotherapy to the facial region in and around the eyelids altered prefrontal hemodynamic responses and autonomic nervous activity during mental arithmetic. Psychophysiology.

[17] Yasui H, Takamoto K, Hori E, Urakawa S, Nagashima Y, Yada Y, Ono T, Nishijo H (2010). Significant correlation between autonomic nervous activity and cerebral hemodynamics during thermotherapy on the neck. Auton Neurosci.

[18] Yoda K, Inaba M, Hamamoto K, Yoda M, Tsuda A, Mori K (2015). Association between poor glycemic control, impaired sleep quality, and increased arterial thickening in Type 2 diabetic patients. PLoS One.

[19] Monoi N, Matsuno A, Nagamori Y, Kimura E, Nakamura Y, Oka K (2016). Japanese sake yeast supplementation improves the quality of sleep: a double-blind randomized controlled clinical trial. J Sleep Res.

[20] Raymann RJEM, Swaab DF, Van Someren EJW (2008). Skin deep: enhanced sleep depth by cutaneous temperature manipulation. Brain.

[21] Nakamura M, Yoda T, Crawshaw LI, Kasuga M, Uchida Y, Tokizawa K (2013). Relative importance of different surface regions for thermal comfort in humans. Eur J Appl Physiol.

[22] Raymann RJ, Swaab DF, Van Someren EJ (2005). Cutaneous warming promotes sleep onset. Am J Physiol Regul integr Comp Physiol.

[23] Borbély AA, Baumann F, Brandeis D, Strauch I, Lehmann D (1981). Sleep deprivation: effect on sleep stages and EEG power density in man. Electroencephalogram Clin Neurophysiol.

[24] Münch M, Knoblauch V, Biatter K, Schröder C, Schnitzier C, Kräuchi K (2004). The frontal predominance in human EEG delta activity after sleep loss decreases with age. Eur J Neurosci.

[25] Anders D, Gompper B, Kräuchi K (2013). A two-night comparison in the sleep laboratory as tool to challenge the relationship between sleep initiation, cardiophysiological and thermoregulatory changes in women with difficulties initiating sleep and thermal discomfort. Physiol Behav.

[26] Kräuchi K (2007). The thermophysiological cascade leading to sleep initiation in relation to phase of entrainment. Sleep Med Rev.

[27] Van Someren EJ (2003). More than a marker: interaction between the circadian regulation of temperature and sleep, age-related changes, and treatment possibilities. Chronobiol int.

[28] Deboer T (1998). Brain temperature dependent changes in the electroencephalogram power spectrum of human and animals. J Sleep Res.

[29] Van Someren EJ (2006). Mechanisms and functions of coupling between sleep and temperature rhythms. Prog Brain Res.

